# Increased Intraspecies Diversity in *Escherichia coli* Biofilms Promotes Cellular Growth at the Expense of Matrix Production

**DOI:** 10.3390/antibiotics9110818

**Published:** 2020-11-17

**Authors:** Andreia S. Azevedo, Gislaine P. Gerola, João Baptista, Carina Almeida, Joana Peres, Filipe J. Mergulhão, Nuno F. Azevedo

**Affiliations:** 1 LEPABE-Laboratory for Process Engineering, Environment, Biotechnology and Energy, Faculty of Engineering, University of Porto, 4200-465 Porto, Portugal; gislainepassarela@hotmail.com (G.P.G.); joaobaptista11023@gmail.com (J.B.); carina.almeida@iniav.pt (C.A.); jperes@fe.up.pt (J.P.); filipem@fe.up.pt (F.J.M.); nazevedo@fe.up.pt (N.F.A.); 2 Laboratório de Investigação em Biofilmes Rosário Oliveira, Centre of Biological Engineering, University of Minho Braga, 4710-057 Braga, Portugal; 3 i3S-Instituto de Investigação e Inovação em Saúde, Universidade do Porto, 4200-135 Porto, Portugal; 4 IPATIMUP-Instituto de Patologia e Imunologia Molecular, Universidade do Porto, 4200-135 Porto, Portugal; 5 INIAV, IP-National Institute for Agrarian and Veterinary Research, Vairão, 4485-655 Vila Do Conde, Portugal

**Keywords:** biofilms, *Escherichia coli*, intraspecies community, EPS matrix, peptide nucleic acid-fluorescence in situ hybridization, urinary tract infections, catheter-associated urinary tract infections, confocal laser scanning microscopy

## Abstract

Intraspecies diversity in biofilm communities is associated with enhanced survival and growth of the individual biofilm populations. Studies on the subject are scarce, namely, when more than three strains are present. Hence, in this study, the influence of intraspecies diversity in biofilm populations composed of up to six different *Escherichia coli* strains isolated from urine was evaluated in conditions mimicking the ones observed in urinary tract infections and catheter-associated urinary tract infections. In general, with the increasing number of strains in a biofilm, an increase in cell cultivability and a decrease in matrix production were observed. For instance, single-strain biofilms produced an average of 73.1 µg·cm^−2^ of extracellular polymeric substances (EPS), while six strains biofilms produced 19.9 µg·cm^−2^. Hence, it appears that increased genotypic diversity in a biofilm leads *E. coli* to direct energy towards the production of its offspring, in detriment of the production of public goods (i.e., matrix components). Apart from ecological implications, these results can be explored as another strategy to reduce the biofilm burden, as a decrease in EPS matrix production may render these intraspecies biofilms more sensitive to antimicrobial agents.

## 1. Introduction

Microorganisms live in a wide variety of environments usually enclosed in communities attached to a surface. This type of community behavior can evolve towards complex multicellular structures, termed biofilms [1,2,3]. These are aggregates of microbial cells embedded in a matrix of extracellular polymeric substances (EPS) that provide several survival advantages, namely, nutrient capture, enzyme retention and resistance/tolerance to antimicrobial agents [4]. Within these structures, bacteria are highly sociable, communicating with each other by secreting important molecules (e.g., cell-signaling molecules, toxins and matrix components), leading to complex interactions during biofilm development [5,6]. This social environment may involve cooperation, competition, or even both.

Microbial competition can manifest as a response to space and nutrient limitation [7,8]. During microbial competition, microorganisms can inhibit the growth of and even kill neighboring cells by secreting broad-spectrum antibiotics [9] or secreting virulent proteins and toxins [10]. However, cooperation is also very prevalent in biofilm communities [11]. For instance, microorganisms can interact to increase their resistance, increasing the tolerance of the whole consortium to antimicrobial agents [12,13], or to enhance the biofilm-forming potential of the consortium [14]. In addition, metabolic cooperation and cross-feeding have been proven to increase the overall fitness of biofilms [15,16]. 

Another interesting theory adapted to the microbial world and, in particular, to the biofilm field, is the “public goods” dilemma [17,18,19]. This theory is based on the observation that, when in society, some bacteria cooperate by secreting a product/resource that becomes available to neighboring bacteria and, hence, benefits the overall consortium. Other bacteria, named cheaters, exploit the “public goods” without contributing for its production, leaving the metabolic burden of synthesizing these molecules to the producing bacteria [20]. Hence, cheaters are likely to outcompete the “public goods” producers and decrease the overall fitness of the consortium [21].

While several examples of cooperation can be found in the biofilm literature for interspecies biofilms, there are far fewer studies with intraspecies biofilms [22,23,24]. Moreover, and to the authors’ knowledge, the number of strains assessed in intraspecies biofilms has always been lower than three. This begs the question: is there a maximum number of strains after which a cooperative behavior in intraspecies biofilms is no longer observed? To answer this question, and as a case study, we formed intraspecies biofilms with up to six strains of *Escherichia coli* isolates from urine, and analyzed their biofilm-forming ability under conditions mimicking the urinary tract infections (UTIs) and catheter-associated urinary tract infections (CAUTIs); UTIs are the most common type of healthcare-associated infection reported. Approximately 75% of hospital-acquired UTIs are associated with CAUTIs [25].

To assess cooperative or competitive behavior, the phylogenetic relatedness between the six *E. coli* strains was investigated and correlated to their performance as biofilm producers. As the self-produced EPS matrix is the most recognizable “public good” under the microbial biofilm context [15], the EPS production was grouped according to the number of strains present in the biofilm, as an indicator of social behavior. Additionally, the spatial location of species within the biofilm architecture was determined by employing a multiplex peptide nucleic acid fluorescence in situ hybridization (PNA-FISH) and confocal laser scanning microscopy (CLSM) analysis.

## 2. Results and Discussion

### 2.1. Single- and Multi-Strain Biofilm Growth

**Figure 1:**
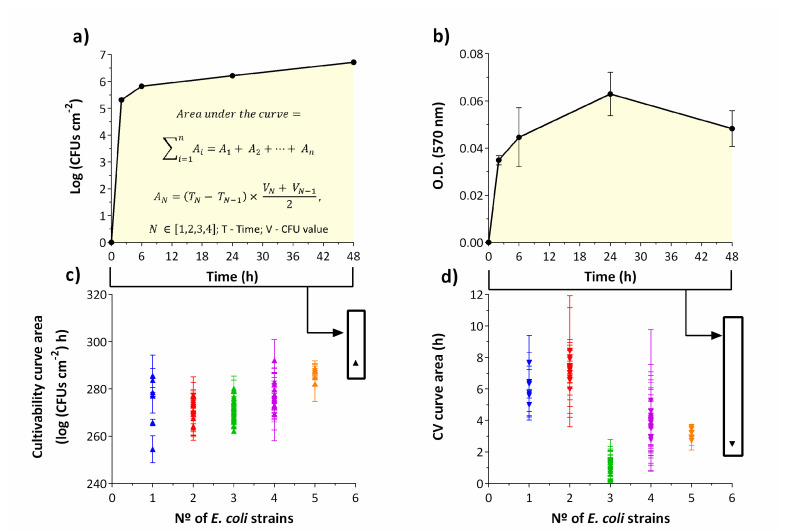
Biofilm formation profiles exhibited by the combination of *Escherichia coli* strains during 48 h, at 37 °C in AUM (artificial urine medium). Example of calculation of the area under the curve for cultivability (**a**) and total biomass (**b**). Cultivability areas showing a relationship between the number of cultivable cells and the number of strains within the biofilm (**c**). CV (crystal violet) areas show a trend of biomass reduction as the number of different strains within the biofilm increases (**d**). The results represent three independent experiments. Results are presented as the mean ± standard deviation. A—partial area; T—time; V—Log (CFUs·cm^−2^) value or O.D.620 nm value.

This work started with the expectation that related strains would cooperate in a biofilm consortium. However, the stage of biofilm development when this behavior, if existent, would occur, was not easily predictable. Moreover, inter-experimental variability in biofilm formation could overshadow the expected changes in metabolism. To increase the chances of picking up this behavior, 63 biofilm growth experiments (Table S1) were conducted, generating an equal number of biofilm growth curves for cultivable cell counts (Figure S1) and total biomass (Figure S2). We then calculated and compared the areas below the curves of the biofilm growth from 0 to 48 h using the trapezium rule [26,27] for both cultivable cell numbers (Figure 1a) and total biomass (Figure 1b).

For all consortia, from 2 h to 48 h, the cultivable cell counts significantly increased over time (*p* < 0.05). In particular, for single-strain biofilms, CFUs counts averaged from 3.6 log CFUs·cm^−2^ at 2 h to 6.5 log CFUs·cm^−2^ at 48 h (*p* < 0.05) (Figure S1a). However, for consortia of 1, 3 and 4 strains there was not a significant difference from 24 h to 48 h in terms of cultivability (*p* > 0.05), i.e., a stationary growth phase seemed to be reached. Concerning the 6 strains biofilm, CFUs counts averaged at 2 h, 5.3 log CFUs·cm^−2^, significantly increased to 6.2 log CFUs·cm^−2^ at 24 h, and 6.7 log CFUs·cm^−2^ at 48 h, (*p* < 0.05) (Figure S1f).

When plotting the number of cultivable cells with the number of strains present in the biofilm (Figure 1c), an increase in the number of strains was accompanied with a slight increase in the number of cultivable cells. Nevertheless, there is no statistically significant difference between the cultivability of 1, 2 and 3 strains consortia (*p* > 0.05). Biofilms composed of 5 and 6 strains are statistically different from 1, 2 and 3 strains consortia (*p* < 0.05). While a slight increase in the number of cultivable cells might have been expected, since the number of cells in the initial suspension also increased with the number of strains, the total produced biomass decreased in general with an increase in the number of strains, particularly for biofilms composed of more than two strains (Figure 1d). In fact, the total biomass significantly decreased when comparing 1 and 2 strains consortia to the remaining biofilms (*p* < 0.05). This behavior is evident comparing the average optical density (O.D._620 nm_) of 0.245 for single-strain and 0.048 for six-strains at 48 h (*p* < 0.05) (Figure S2). Still, an unexpected higher decrease was obtained for biofilms composed of three strains, not confirmed in later EPS quantification, which can evidence that loosely attached biofilm could have been dragged out during washing steps (i.e., EPS production still occurred but the physico-chemical forces of attachment to the surface was weaker).

For biofilms up to 5 strains, from 2 h to 48 h, the produced biomass significantly increased with time (*p* < 0.05) (Figure S2). However, when six strains were present, a significant increase in biomass production over time was not observed (*p* > 0.05) (Figure S2f). In addition, the biomass seemed to decrease for the 3 strains consortia during the incubation period (*p* < 0.05) (Figure S2c).

### 2.2. Biomass and Matrix Production as a Function of the Number of Strains in a Consortium

**Figure 2:**
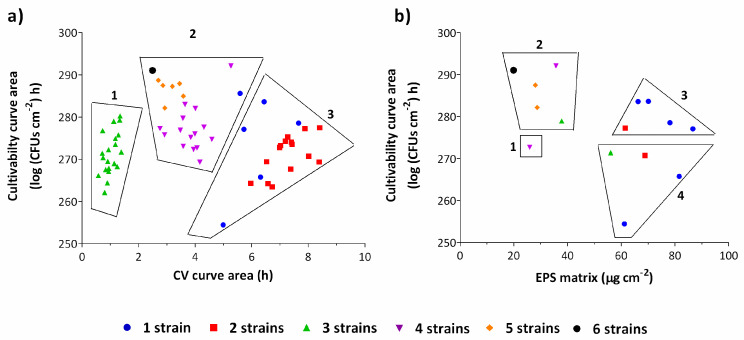
Cluster analysis for cultivability areas versus CV areas (**a**) and for cultivability areas versus EPS (extracellular polymeric substances) matrix (**b**) for 48 h-aged biofilms. Clusters are observable according to the number of strains in consortia. A trend for multi-strain biofilms to produce less EPS matrix is noticeable. The graph (**a**) is subdivided in three clusters: low cell numbers and biomass (1); high cell numbers and low biomass (2); low cell numbers and high biomass (3). The graph (**b**) is subdivided in four clusters: low cell numbers and EPS matrix (1); high cell numbers and low EPS matrix (2); high cell numbers and EPS matrix (3) and low cell numbers and high EPS matrix (4). The results represent three independent experiments. Results are presented as the mean.

A cluster analysis (Figure 2) was conducted to provide a statistical basis on the observation that an increasing number of strains in a biofilm would affect its behavior, plotting the cultivable cell counts versus total biomass (Figure 2a) and versus EPS matrix (Figure 2b). Overall, a higher number of strains led to a lower amount of biomass production (*p* < 0.05). In particular, two strains biofilms were mainly clustered as low cell numbers and high biomass producers. Biofilms composed by three strains were clustered as very low biomass producers, contradicting the overall behavior. Biofilms composed by 4, 5 and 6 strains were clustered as high cell numbers and low biomass producers (Figure 2a).

To confirm these clusters, the EPS matrix was quantified for a subset of biofilms. Analyzing the clusters (Figure 2b), there is a tendency for multi-strain biofilms to produce less EPS matrix than single-strain biofilms. Biofilms composed by one and two strains were clustered as high EPS producers while biofilms with 4, 5 and 6 strains grouped as high cell numbers and low EPS producers. In fact, the produced EPS matrix decreased almost linearly with the increase in the number of strains in the consortia (Figure S3). Interestingly, for biofilms composed of three strains, the above-mentioned low biomass production was not confirmed by EPS matrix quantification by dry weight after lyophilization (Figure 2b). 

Overall, this work shows that an increase in the number of *E. coli* strains in an intraspecies biofilm redirects the metabolism of the microorganisms towards offspring production at the expense of EPS matrix. However, more work is required in the future with other relevant bacterial species and other biofilm growth conditions (e.g., media culture, surfaces, hydrodynamic conditions) to investigate if the observed intraspecies phenomenon presented in this study is maintained. 

### 2.3. How Long Does It Take for Microorganisms to Adapt Their Metabolism?

**Figure 3:**
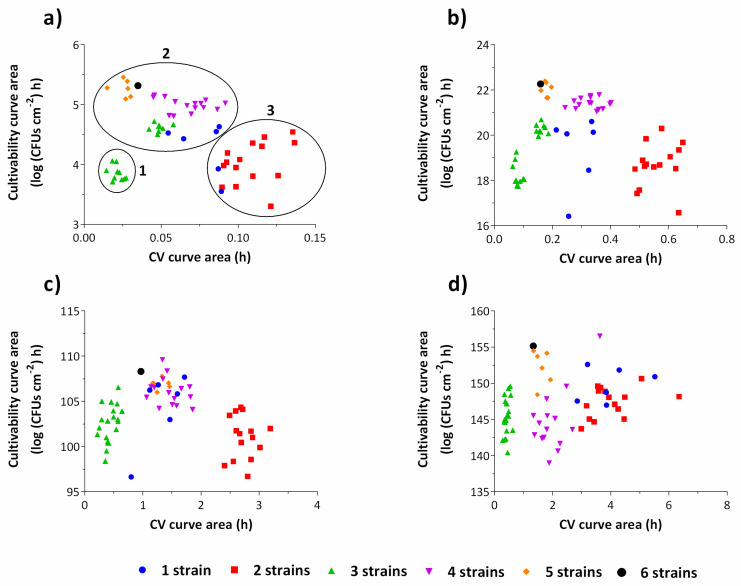
Cluster analysis for cultivability areas versus CV areas for each time point of the experiments: 0–2 h (**a**), 2–6 h (**b**), 6–24 h (**c**) and 24–48 h (**d**). The clustering phenomenon is more evident during the first hours of biofilm development, namely up to 24 h of growth. The graph (**a**) is subdivided in three clusters: low cell numbers and biomass (1); high cell numbers and low biomass (2); low cell numbers and high biomass (3). The results represent the mean of three independent experiments.

To better understand when the metabolism of the microorganisms is directed towards cell growth rather than EPS matrix production during biofilm growth, a cluster analysis for each of the time intervals of the experiment was performed (Figure 3). It is clear that, for 2 h-old biofilms, the trend for multi-strain biofilms to produce less biomass can already be observed (Figure 3a). 

In fact, as the biofilm matures, the biofilm clustering according to the number of strains starts to be less evident (Figure 3c,d). After two hours of biofilm growth, two strains biofilms were clustered as low cell numbers and high biomass producers, while biofilms composed by 4, 5 and 6 strains and most of the biofilms composed by 1 strain were grouped as high cell numbers and low biomass producers. This behavior is maintained for the subsequent time points, with the exception of most of the biofilms composed by 4 strains which present low cell numbers after 48 h of growth. Interestingly, after 2 h, some of the three strains biofilms are grouped together with 4 strains biofilms. It is only afterwards that they develop the less expected behavior of very low biomass production. These results indicate that the differentiated behavior occurs predominantly within the first hours of biofilm development, which is expected. In fact, *E. coli* is known to rapidly direct its metabolism when adapting to diverse and sudden stress conditions [28,29]. In a study of Drazic et al. [30], major changes in the metabolic profile of *E. coli* occurred as early as after 5 min of hypochlorite-induced stress, in terms of relative concentrations of fatty acids, amino acids, acetic and formic acid, which were readily regenerated after 40 to 60 min.

### 2.4. Impact of Phylogenetic Closeness in Biomass Production for Two-Strain Biofilms

In theory, the microbial cooperation is promoted by high relatedness of microbial cells. To infer on this, seven housekeeping genes (*adk*, *fumC*, *gyrB*, *icd*, *mdh*, *purA* and *recA*) were sequenced in order to infer whether genetic similarity among the strains would impact the offspring and total biomass production of the overall consortia, according to the ∑ Cb_I_. According to this analysis, the percentage of different nucleotides among these isolates is below 1.8%. In a study of Lukjancenko et al. [31], the highest phylogenetic difference obtained, when comparing several *E. coli* strains using the same housekeeping genes, was around 2%, which seems to be in agreement with our findings.

**Figure 4:**
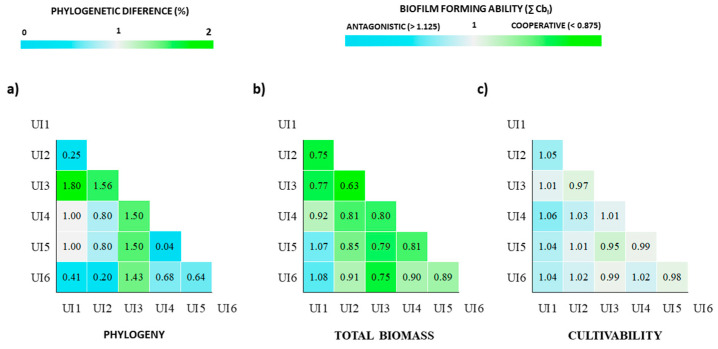
Heat maps showing the phylogenetic differences between the *E. coli* urinary isolates in percentage (**a**) and their biofilm-forming ability when combined with each other in pairs (**b**,**c**). Phylogenetic percentages were calculated using the different nucleotides in the 7 housekeeping genes between the six *E. coli* strains, based in the neighbor-joining method and pairwise distance. The biofilm-forming ability of the combined consortia was scored in terms of total produced biomass (**b**) and cultivability (**c**) according to the ∑ Combinatorial biofilm index (∑ Cb_I_ scoring: cooperative (<0.875), neutral (0.875 < ∑ Cb_I_ < 1.125) and antagonistic (>1.125)] for 0–48 h period of biofilm formation. This analysis on total biomass suggests that both high and low phylogenetic distance between the strains can be accompanied with an increase in the produced biomass, namely, when UI3, the most distant, was paired with all the remaining, as well as when related strains (UI1 with UI2; UI4 with UI5) were combined. All the pairs in terms of cultivability were scored as neutral.

Due to the lack of adequate methods to statistically assess the phylogenetic closeness impact of several strains in biofilm formation, only two-strain biofilms were analyzed. Urinary isolates (UI) 1, UI2 and UI6 present very low genetic variability between each other. UI4 and UI5 are also genetically similar. UI3 is the most genetically distant within these strains of *E. coli* (Figure 4a). The impact of the phylogeny was then assessed in terms of biofilm biomass (Figure 4b) and cell production (Figure 4c). 

The biomass and cultivability of the biofilms were scored as cooperative, neutral or antagonistic according to the ∑ Combinatorial biofilm index (∑ Cb_I_). Interestingly, in terms of total biomass, all the combinations of the UI3 (the most distant) with the remaining were scored as cooperative (0.63 < ∑ Cb_I_ < 0.80). Still, the combinations of related strains such as UI1 with UI2 as well as UI4 with UI5 were also scored as cooperative. This analysis suggests that both high and low phylogenetic distance between the strains can be accompanied with an increase in the produced biomass. A neutral score is obtained when analyzing the ∑ Cbi for all the consortia in terms of cultivability (Figure 4c). From this analysis, it appears that for two-strains biofilms, the phylogenetic relatedness is inversely proportional to the biomass production ability of the two-strain consortium. An open question remains on whether this behavior will still be observed when more than two-strain biofilms are analyzed in the future. 

### 2.5. Spatial Organization of Biofilms Using PNA-FISH Combined with CLSM

It has been demonstrated that microorganisms locate and organize themselves within biofilms according to the nature of their microbial interactions. In general, microorganisms organize in three main forms: a segregation form associated with competition, a co-aggregation/intermixing structure related with cooperation and a layering arrangement found in cooperative or competitive behavior [32]. 

**Figure 5:**
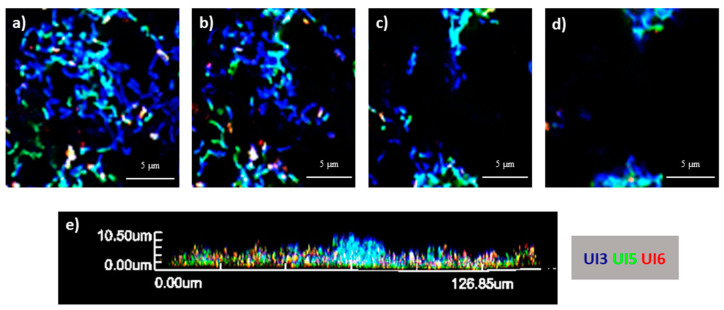
Three-dimensional organization of 6 h-aged biofilm formed in AUM and in polystyrene coupons by a consortium of three *E. coli* strains (UI3—blue, UI5—green and UI6—red). (**a**) Examples of CLSM (confocal laser scanning microscopy) images obtained of the layers within the biofilm at different heights (**a** = 0 μm; **b** = 0.7 μm; **c** = 2.1 μm; **d** = 3.5 μm). (**e**) Cross-section of the biofilm.

In the present study, to better understand the behavior between the strains, a multiplex PNA-FISH combined with CLSM was performed for the analysis of the spatial distribution of *E. coli* multi-strain biofilms. First, the hybridization of the PNA probes was previously optimized in terms of temperature and formamide concentration. Optimal conditions for multiplex FISH were obtained at 50 °C and 30% (*v*/*v*) formamide. PNA-UI126 was specific for UI2 and 6. However, probe PNA-UI5 exhibited a certain level of fluorescence for the remaining strains when alone. Nevertheless, this can be resolved by multiplex FISH analysis, making it possible to identify a consortium composed by three strains (Figure S4). The CLSM 3D images of the morphology of the biofilms, z planes and their cross-section are presented in Figure 5, Figure S5 and Figure S6 for 6 h, 24 h and 48 h of biofilm growth, respectively. Overall, the tested consortia showed, in general, a coaggregation structure. Concerning biofilms incubated for 6 h, aggregates could be found, such as the one at the center of Figure 5e, composed mainly by strains 3 and 5. In fact, the strains seem to be well mixed and aggregated to each other. Similar findings were obtained when *E. coli* is present alone in consortium with other species [33,34]. 

Intraspecies coaggregation is stated as important for the development of biofilms, enabling metabolic interactions, cell–cell communication and genetic exchange [35]. Therefore, we theorize that the strains can co-exist in the same space and are just enough far apart phylogenetically to cooperate when intraspecies diversity is increased in the surroundings. To understand this observed intraspecies phenomenon and to explain why an increase in diversity alters the EPS/cell numbers on these biofilms, we suggest that an intraspecies facultative cooperation strategy occurs, allowing them to save energy or to share resources. On the other hand, the proximity of the strains within the consortia may also facilitate the exchange of genes that confer advantages or the accumulation of mutations, helping them to adapt to the environmental conditions when the number of strains in a consortium is increased.

## 3. Materials and Methods

### 3.1. Bacterial Strains and Growth Conditions

In this study, six different *E. coli* strains were isolated from urine samples of patients using Cystine lactose electrolyte deficient agar (CLED) medium and MacConkey agar medium (Liofilchem, Roseto degli Abruzzi, Italy). Agar plates were incubated for 24 h at 37 °C. The identity of the six *E. coli* isolates was confirmed by sequencing the 16S ribosomal RNA (16S rRNA) gene, performed by StabVida, Lda (Caparica, Lisbon), and later confirmed using the Basic Local Alignment Search Tool (BLAST; https://blast.ncbi.nlm.nih.gov). The *E. coli* strains were named urinary isolates (UI) followed by a number from 1 to 6 (e.g., UI1).

For each experiment, isolates were recovered from −80 °C glycerol stock cultures on Tryptic Soy Agar (TSA) (Merck, Darmstadt, Germany) and grown for 24 h at 37 °C.

### 3.2. Single- and Multi-Strain Biofilms Assays

For the preparation of each inoculum, isolated colonies for each strain were inoculated into artificial urine medium (AUM) [36] and incubated overnight (16–18 h), at 37 °C and 150 rpm. Subsequently, cell concentration was assessed by optical density at 620 nm (O.D._620 nm_) and each inoculum was diluted in AUM in order to obtain a cell concentration of 10^6^ CFUs·mL^−1^.

In order to evaluate the biofilm-forming ability of each strain and in consortium, single- and multi-strain biofilms were grown as previously described [37]. Briefly, 200 µL of each inoculum prepared in AUM (10^6^ CFUs·mL^−1^) were added to each well of a 96-well tissue culture plate (Orange Scientific, Braine-l’Alleud, Belgium). Regarding multi-strain biofilms, an adequate volume of each strain culture was mixed, keeping a final volume of 200 µL with an initial concentration of 10^6^ CFUs·mL^−1^ for each strain and added to each well. The plates were incubated at 37 °C, under static conditions, for 48 h. At predetermined time points (2, 6, 24 and 48 h), the biofilms were washed with 200 µL of 0.85 % (*w*/*v*) sterile saline solution to remove non-adherent and loosely attached cells. Then, biofilm formation was assessed by crystal violet (CV) staining (for total biomass quantification) and colony forming units (CFUs) counts (for cultivable cell counts). Quantification of extracellular polymeric matrix of single- and multi-strain biofilm was also performed for 48 h biofilms. These assays were performed with two or more independent experiments. Tested combinations are shown in Table S1 in Supplementary Information.

### 3.3. Biomass Quantification by Crystal Violet Staining

The produced biomass by single- and multi-strain biofilms in 96-well tissue culture plate was assessed by the CV staining [38]. The washed biofilms were fixed with 250 µL of 99% ethanol (*v*/*v*) for 15 min. Then, ethanol was removed, and the plates air-dried. Subsequently, biofilms were stained with 250 µL of CV (Merck, Germany) for 5 min. Microplates were rinsed with water, air-dried and the CV was resolubilized by adding 200 µL of 33 % (*v*/*v*) glacial acetic acid (Merck, Germany) to each well. Plates were stirred for 2 min and the content was transferred to new 96-well plates for O.D. (570 nm) measure using a microtiter plate reader (Spectra Max M2, Molecular Devices, Sunnyvale, CA, USA).

### 3.4. Cultivability Assessment

The number of cultivable biofilm cells was determined by CFUs counting as previously described by Azevedo, Almeida, Melo and Azevedo [37]. Briefly, after the washing step, 200 µL of 0.85 % (*w*/*v*) sterile saline solution were added into each well containing the biofilms. Biofilms were sonicated for 4 min (70 W, 35 kHz, Ultrasonic Bath T420, Elma, Singen, Germany) and 100 µL of the disrupted biofilms were serially diluted (1:10) in 0.85% (*w*/*v*) sterile saline solution and plated in triplicate in TSA plates (sonication conditions were previously optimized by Azevedo et al. [34]. The TSA plates were incubated at 37 °C for 14 h. The number of CFUs was expressed in logarithm per microtiter plate well’s bottom and side area (log CFUs·cm^−2^).

### 3.5. EPS Matrix Quantification by Dry Weight

EPS quantification was performed to a subset of biofilms (P1, P2, P3, P4, P5 and P6 (1 strain); P7 and P21 (2 strains); P22 and P41 (3 strains); P42 and P56 (4 strains); P57 and P62 (5 strains); P63 (6 strains)). For that, an extraction procedure was applied to separate the exopolymeric substances from the microbial cells [39,40]. The washed biofilms were sonicated in an ultrasonic bath for 4 min (70 W, 35 kHz, Ultrasonic Bath T420, Elma, Singen, Germany). The bacterial suspensions were transferred to universal tubes (50 mL) and sonicated (10 s, 25% amplitude). The bacterial suspensions were vortexed for 2 min and centrifuged for 10 min at 3000 rpm (at 4 °C). Then, the supernatants were filtered through a membrane (0.2 µm), using a syringe, to pre-weighed tubes. The tubes were frozen at −80 °C during 48 h for liquid extraction by lyophilization. After lyophilization, the tubes were weighed, and the total mass of the EPS matrix was determined.

### 3.6. Multilocus Sequence Typing for Phylogenetic Analysis

Multilocus Sequence Typing (MLST), as previously described by Liu et al. [41], was used to determine the diversity and phylogenetic relationships of the six *E. coli* strains. The sequencing of seven housekeeping genes, *adk* (Adenylate kinase), *fumC* (Fumarate hydratase), *gyrB* (DNA gyrase subunit B), *icd* (Isocitrate dehydrogenase), *mdh* (Malate dehydrogenase), *purA* (Adenylosuccinate synthetase) and *recA* (recA protein, repair and maintenance of DNA), was performed by StabVida, Lda (Caparica, Lisbon) following the protocols specified at the *E. coli* MLST website (http://mlst.warwick.ac.uk/mlst/dbs/Ecoli). For each obtained DNA sequence, the consensus sequence was generated after all gaps and single nucleotide changes were checked in the chromatograms for forward and reverse sequences using the Geneious 9.0.4 software (Biomatters Limited, New Zealand). Afterwards, sequences of all seven genes were concatenated for each isolate and aligned using Geneious 9.0.4 software. The phylogenetic analysis was inferred by the neighbor-joining algorithm using Geneious 9.0.4 software for the calculation of the pairwise distances in percentage [42].

### 3.7. Impact of the Phylogenetic Closeness in Biomass Production

In order to evaluate the phylogenetic impact in the biofilm development when the referred strains were combined, a scoring method was applied to the CV and CFUs areas (48 h growth) obtained for all the combinations. Therefore, the biofilm-forming ability and cultivability of the *E. coli* strains were scored according to the ∑ Combinatorial biofilm index (∑ Cb_I_) adapted from Baptista et al. [43] presented in Equations (1) and (2):

(1) $$Cb_{I\left(N\right)}=\;\frac{Curve\;Area_{\left(N\right)}}{Curve\;Area_{\left(Combined\right)}},\;N\;\in \left[1,2,3,4,5,6\right]$$

where *Cb_I_* is the *Cb* index, which is calculated for each strain to be compared, dividing the curve area (total biomass or cultivability) of each strain for the curve area when those strains are combined. Then, the sum of the *Cb_I_* for each strain in consortia gives the ∑ Cb_I_ as exemplified in Equation (2):

(2) $$\sum_{i=1}^{n}Cb_{I\left(i\right)}=\left(Cb_{I\left(1\right)}+\;Cb_{I\left(2\right)}+\ldots +\;Cb_{I\left(n\right)}\right)/n$$

The obtained values for ∑ Cb_I_ are then scored according to the following criteria: <0.875—Cooperative; 0.875 to 1.125—Neutral; >1.125—Antagonistic. In addition, heat maps were constructed to better infer about the impact of the phylogenetic distance between the strains.

### 3.8. Optimization of the PNA-FISH Protocol

Two PNA-FISH probes were designed to target the *E. coli* strains (one probe for UI1, UI2 and UI6 strains; and another one for UI5 strain). Mismatches in the 16S rRNA sequences between the six *E. coli* strains were analyzed using Clustal Omega multiple sequence alignment tool (https://www.ebi.ac.uk/Tools/msa/clustalo/). Probes were selected according to their base pair (bp) length, mismatch localization, Guanine/Cytosine (GC) content, theoretical melting temperature point (T_m_) and Gibbs free energy (ΔG < −13) [27] (Table 1). The probes were attached to Alexa Fluor 488 (PNA-UI5) and Alexa Fluor 594 (PNA-UI126) signaling molecules via a double 8-amino-3, 6-dioxaoctanoic acid (AEEA) linker (Panagene, Daejeon, South Korea, HPLC purified > 90%). For UI3 and UI4 strains, 4′-6-Diamidino-2-phenylindole (DAPI; Merck, Germany) was used as a counterstain.

UI5 and UI2 strains were selected to optimize the hybridization conditions of the two PNA-FISH probes. A range of temperature and formamide concentrations were evaluated for a better microscopic signal using a LEICA DMLB2 epifluorescence microscope (Leica Microsystems Ltd.; Wetzlar, Germany) coupled with a Leica DFC300 FX camera (Leica Microsystems Ltd.; Wetzlar, Germany), with 100× oil immersion fluorescence objective. Images were acquired using Leica IM50 Image Manager, Image processing and Archiving software. The hybridization procedure in microscope slides was performed according to Almeida et al. [44]. Briefly, smears (30 μL) of each bacterial strain (OD_620nm_ = 0.1 ≈ 10^8^ cells·mL^−1^) in sterile distillate water were applied in microscope slides and immersed in 4% paraformaldehyde (30 μL) (Sigma-Aldrich, St. Louis, MO, USA) for 15 min. Then, 30 μL of 50% ethanol were added to the smears for 15 min and air-dried. Afterwards, the smears were covered with 20 μL of hybridization solution containing 10% dextran sulphate (Sigma-Aldrich, St. Louis, MO, USA), 10 mM NaCl (Sigma-Aldrich, St. Louis, MO, USA), 0.1% (*wt*/*vol*) sodium pyrophosphate (Sigma-Aldrich, St. Louis, MO, USA), 0.2% (*wt*/*vol*) polyvinylpyrrolidone (Sigma-Aldrich, St. Louis, MO, USA), 0.2% (*wt*/*vol*) Ficol (Sigma-Aldrich, St. Louis, MO, USA), 5 mM disodium EDTA (Sigma-Aldrich, St. Louis, MO, USA), 0.1% (*vol*/*vol*) Triton X-100 (Sigma-Aldrich, St. Louis, MO, USA), 50 mM Tris-HCl (Sigma-Aldrich, St. Louis, MO, USA), 30% or 50% (*vol*/*vol*) of formamide (Acros Organics, Belgium), and 200 nM of PNA probe. Smears with hybridization solution without PNA probe were performed as negative controls. Samples were covered with coverslips and placed in an incubator for a range of temperatures (48 °C to 54 °C) for 90 min. Then, the microscope slides were immersed in a pre-warmed washing solution containing 5 mM Tris base (Sigma-Aldrich, St. Louis, MO, USA), 15 mM NaCl (Sigma-Aldrich, St. Louis, MO, USA) and 1% (*vol*/*vol*) Triton X (pH = 10, Sigma-Aldrich, St. Louis, MO, USA), without their coverslips for 30 min at the same temperature of the hybridization step. The smears were covered with a drop of non-fluorescent immersion oil (Merck, Germany) and watched at the microscope. The microscope slides were stored for a maximum of 24 h in the dark at 4 °C before microscopy.

### 3.9. Study of the Spatial Organization in E. coli Multi-Strain Biofilms Using PNA-FISH Combined with Confocal Laser Scanning Microscopy Analysis

In order to assess the biofilm spatial organization and the strains distribution, the PNA-FISH and DAPI staining were performed directly in biofilms formed in polystyrene coupons as previously described [27,33]. Briefly, the biofilm formation was conducted as previously described. Then, 3 mL of the bacterial suspensions were added to each well of 12-well microtiter plates, with previously- placed sterilized polystyrene coupons (prepared according to Azevedo et al. [45]) at the bottom. The plates were then incubated for 6 h, 24 h and 48 h, under static conditions. After the incubation period, the coupons were carefully transferred and washed in another sterile 12-well microtiter plates with 3 mL of 0.85% (*w*/*v*) sterile saline solution and dried for 15 min at 60 °C. Afterwards, the biofilms in the coupons were placed carefully in a microscope slide, and the hybridization was performed as previously described for 90 min at 50 °C. When UI3 and UI4 were present, DAPI staining was performed. For this, a drop of DAPI solution (0.1 mg·mL^−1^; Merck, Germany) was added to the coupons for 10 min in the dark at room temperature. 

After the FISH procedure, the analysis of multi-strain biofilms structure and the location of the bacterial strains was made using a confocal laser scanning microscopy (Olympus BX61, Model FluoView 1000) and the multichannel simulated fluorescence projection of images and vertical cross-sections through the biofilm were generated by using the FluoView 1000 Software package (Olympus). During the analysis, a 60× water-immersion objective (60×/1.2 W) was used.

A laser excitation line 405 nm and emission filters BA 430-470 (blue channel) were used for DAPI observation; PNA-UI5 probe coupled to Alexa Fluor 488 was observed using a laser excitation line 488 nm and emission filters BA 505-540 (green channel); and for observation of PNA-UI126 probe coupled to Alexa Fluor 594, a laser excitation line 559 nm and emission filters BA 575-675 (red channel) was used. 

### 3.10. Statistical Analysis

The results were compared using one-way analysis of variance (ANOVA) by applying the Tukey multiple-comparisons post-hoc test, using the Statistical Package for the Social Sciences (SPSS) Statistics 25 (IBM, Armonk, NY, USA). All tests were performed with a confidence level of 95%. The results were also compared by model-based clustering, using R software. The standard methodology selects the number of clusters according to the Bayesian information criterion (BIC) [46,47].

## 4. Conclusions

In conclusion, these results may represent another way to fight biofilm-related infections. We show that even in fully functional microorganisms, cells are induced to produce less EPS matrix when other strains are present. Nonetheless, most clinical biofilms are thought to be caused by a single strain. Hence, introducing multiple avirulent strains of the infecting microorganism at the site of infection, we might be inducing an EPS matrix-deficient biofilm which is, in theory, more susceptible to antibiotics/antimicrobial treatment. Moreover, as most of the antibiotics are most active in dividing cells [48], promoting cellular growth while increasing diversity, we might also increase susceptibility to antibiotics. For such strategy to work broadly, this approach must be applicable to intraspecies biofilms of other species and to pre-formed biofilms. Future lines of work will also include testing of these multi-strain biofilms in the presence of currently used antibiotics.

## Figures and Tables

**Table 1:** Sequences of the PNA-FISH probes, their target positions in the 16S rRNA and theoretical values including bp (base pair), % GC, ΔG, and T_m_.

PNA-Probes	Strains	Sequence	Position in 16S rRNA	bp	% GC	ΔG (kcal/mol)	T_m_ (°C)
PNA-UI126	UI1, 2 and 6	5′-GTGAGCCTTTACCC-3′	144 to 157	14	0.57	−16.43	73.30
PNA-UI5	UI5	5′-TCCATCGGGCAGT-3′	18 to 30	13	0.62	−15.88	75.96

**_**—mismatch position.

## Supplementary Materials

The following are available online at https://www.mdpi.com/2079-6382/9/11/818/s1, Figure S1: Number of cultivable cells in single-strain biofilms and in multi-strain biofilms combining two, three, four, five and six *E. coli* strains, during 48 h. Standard deviations of three independent replicates are displayed. Figure S2. O.D. values for total biomass quantification (CV method) for single-strain biofilms and for multi- strain biofilms combining two, three, four, five and six *E. coli* strains, during 48 h. Standard deviations of three independent replicates are displayed; Figure S3. Mean EPS matrix concentration ± standard deviation of biofilms formed by consortia of 1 up to 6 different *E. coli* strains after 48 h of incubation at 37 °C in AUM. The produced EPS matrix decreases linearly with the addition of strains in consortia, adjusted with an R-squared of 0.94. Figure S4. Multiplex FISH using both PNA-UI5 and PNA-UI126 probes using UI5 and UI2, respectively, at 50 °C hybridization temperature and 30% formamide concentration. a–Green filter; b–Red filter; c–Filters overlapping. A magnification of 1500× was used. Figure S5. Three-dimensional organization of 24 h aged biofilm formed in AUM and in polystyrene coupons by a consortium of three *E. coli* strains (UI3-blue, UI5-green and UI6-red). (a) Examples of CLSM images obtained of the layers within the biofilm at different heights (a = 0 μm; b = 1 μm; c = 2 μm; d = 3 μm). (e) Cross section of the biofilm. Figure S6. Tri-dimensional organization of 48 h aged biofilm formed in AUM and in polystyrene coupons by a consortium of three *E. coli* strains (UI3-blue, UI5-green and UI6-red). (a) Examples of CLSM images obtained of the layers within the biofilm at different heights (a = 0 μm; b = 2 μm; c = 4 μm; d = 6 μm). (e) Cross section of the biofilm. Table S1: Number of possible combinations (P) of the six different *E. coli* strains used for biofilms formation assays.
