# Supplementary material for: Increased Intraspecies Diversity in Escherichia coli Biofilms Promotes Cellular Growth at the Expense of Matrix Production
Source: Antibiotics (Basel). 2020 Nov 17;9(11):818. doi: 10.3390/antibiotics9110818 (PMC7698454; doi:10.3390/antibiotics9110818)
Supplement: Supplementary file 1 [file antibiotics-09-00818-s001.pdf]

Supplemental material

Tables

Table S1. Number of possible combinations (P) of the six different *E. coli* strains used for biofilms formation assays.

	Number of strains in the consortium					
	One strain	Two strains	Three strains	Four strains	Five strains	Six strains
Possible combinations (P)	P1 – UI1	P7 – UI1;2	P22 – UI1;2;3	P42 – UI1;2;3;4	P57 – UI1;2;3;4;5	P63 – UI1;2;3;4;5;6
	P2 – UI2	P8 – UI1;3	P23 – UI1;2;4	P43 – UI1;2;3;5	P58 – UI1;2;3;4;6	
	P3 – UI3	P9 – UI1;4	P24 – UI1;2;5	P44 – UI1;2;3;6	P59 – UI1;2;3;5;6	
	P4 – UI4	P10 – UI1;5	P25 – UI1;2;6	P45 – UI1;3;4;5	P60 – UI1;2;4;5;6	
	P5 – UI5	P11 – UI1;6	P26 – UI1;3;4	P46 – UI1;3;4;6	P61 – UI1;3;4;5;6	
	P6 – UI6	P12 – UI2;3	P27 – UI1;3;5	P47 – UI1;3;5;6	P62 – UI2;3;4;5;6	
		P13 – UI2;4	P28 – UI1;3;6	P48 – UI1;4;5;6		
		P14 – UI2;5	P29 – UI1;4;5	P49 – UI1;2;5;6		
		P15 – UI2;6	P30 – UI1;4;6	P50 – UI1;2;4;5		
		P16 – UI3;4	P31 – UI1;5;6	P51 – UI1;2;4;6		
		P17 – UI3;5	P32 – UI2;3;4;	P52 – UI2;3;4;5		
		P18 – UI3;6	P33 – UI2;3;5	P53 – UI2;3;4;6		
		P19 – UI4;5	P34 – UI2;3;6	P54 – UI2;4;5;6		
		P20 – UI4;6	P35 – UI2;4;5	P55 – UI3;4;5;6		
		P21 – UI5;6	P36 – UI2;4;6	P56 – UI2;3;5;6		
			P37 – UI2;5;6			
			P38 – UI3;4;5			
			P39 – UI3;4;6			
			P40 – UI3;5;6			
			P41 – UI4;5;6			

Figures

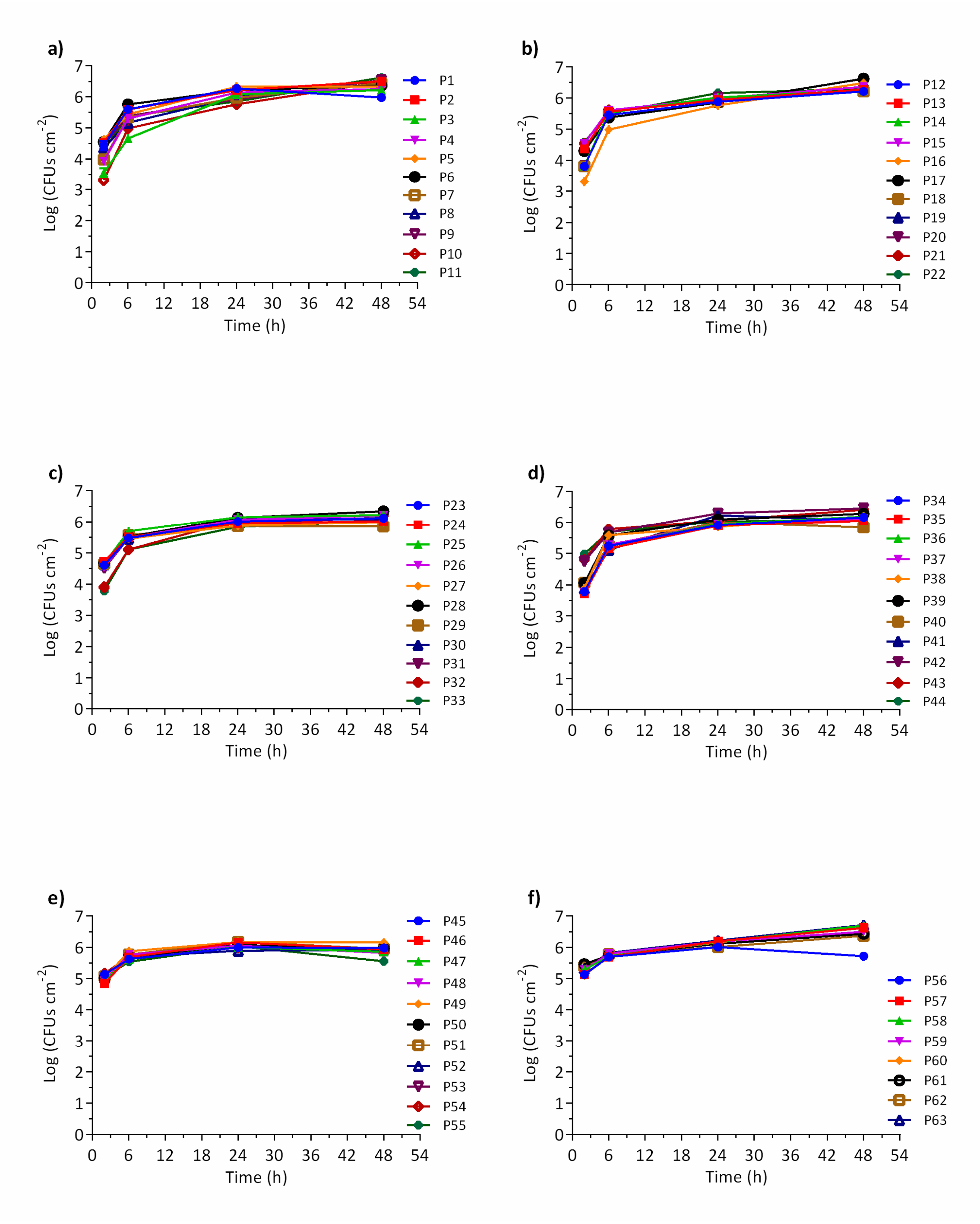

Figure S1. Number of cultivable cells in single-strain biofilms and in multi-strain biofilms combining two, three, four, five and six *E. coli* strains, during 48 h. Standard deviations of three independent replicates are displayed.

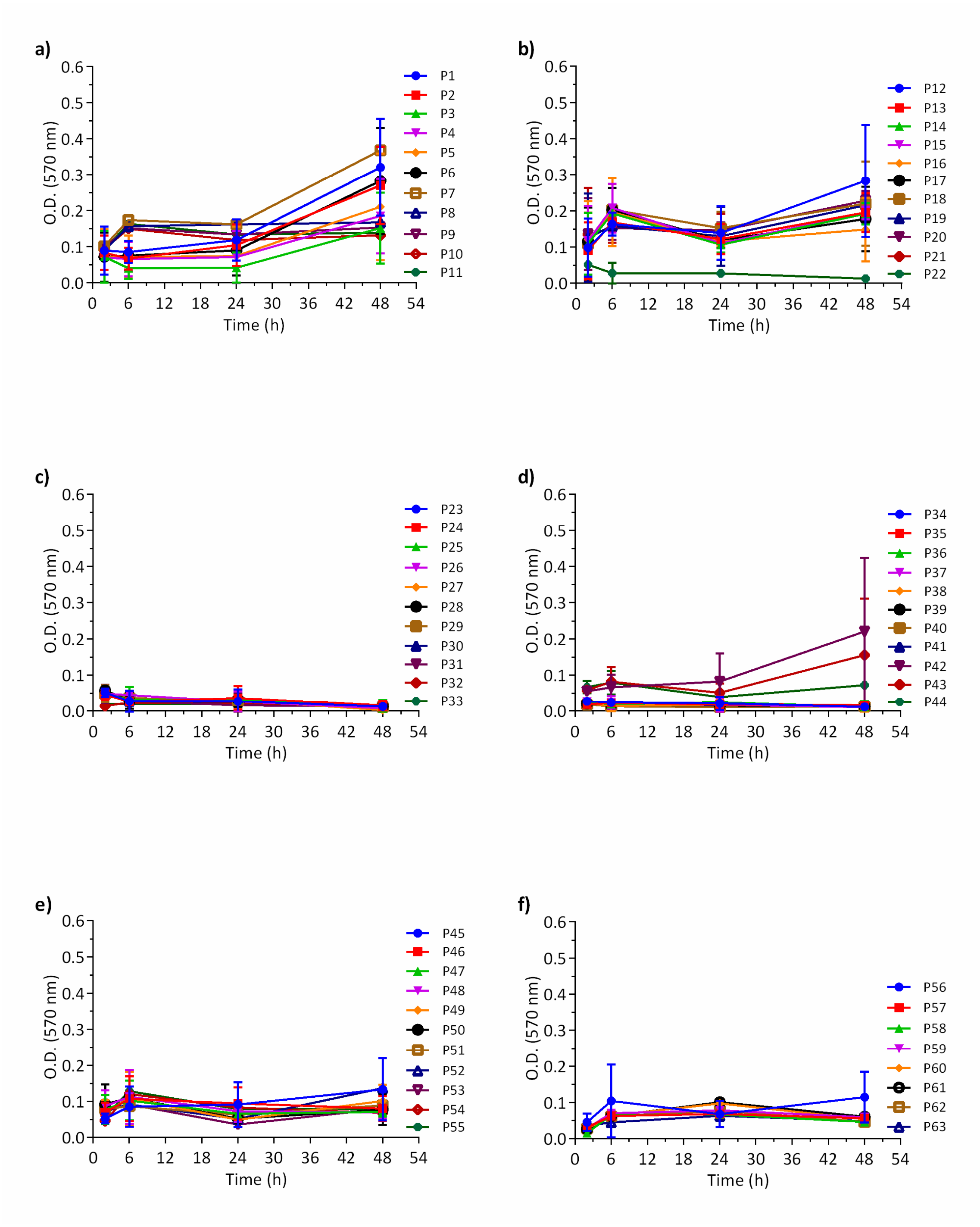

Figure S2. O.D. values for total biomass quantification (CV method) for single-strain biofilms and for multi-strain biofilms combining two, three, four, five and six *E. coli* strains, during 48 h. Standard deviations of three independent replicates are displayed.

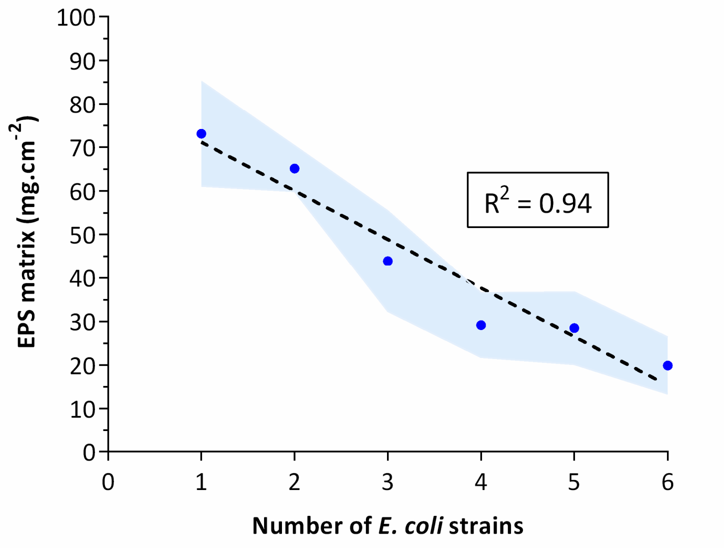

Figure S3. Mean EPS matrix concentration ± standard deviation of biofilms formed by consortia of 1 up to 6 different *E. coli* strains after 48 h of incubation at 37 ºC in AUM. The produced EPS matrix decreases linearly with the addition of strains in consortia, adjusted with an R-squared of 0.94.

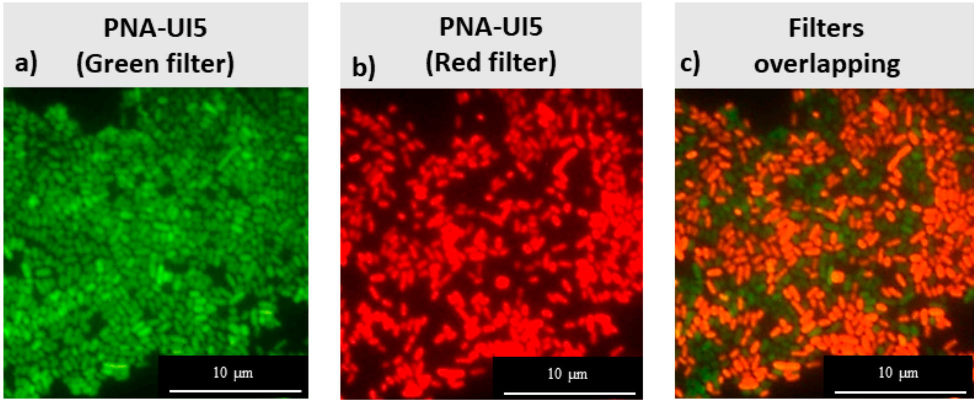

Figure S4. Multiplex FISH using both PNA-UI5 and PNA-UI126 probes using UI5 and UI2, respectively, at 50 °C hybridization temperature and 30 % formamide concentration. a – Green filter; b – Red filter; c – Filters overlapping. A magnification of 1500 × was used.

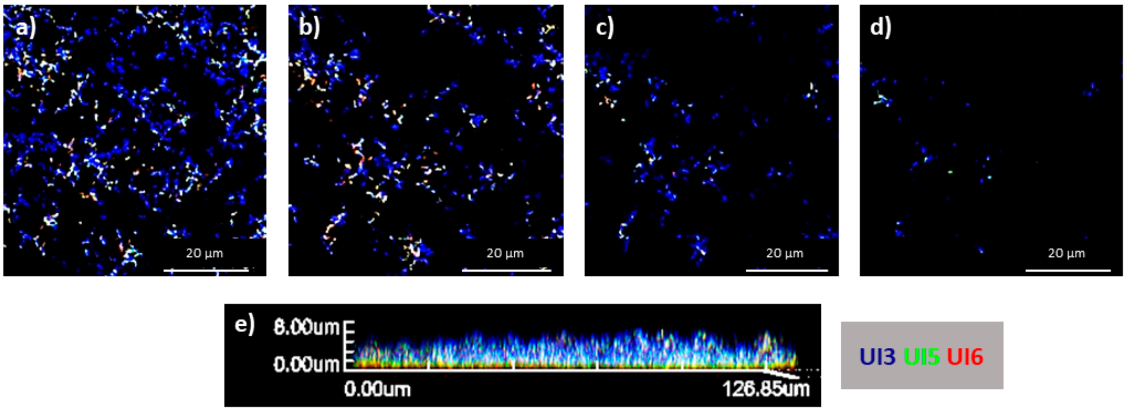

Figure S5. Three-dimensional organization of 24 h aged biofilm formed in AUM and in polystyrene coupons by a consortium of three *E. coli* strains (UI3 - blue, UI5 - green and UI6 - red). (a) Examples of CLSM images obtained of the layers within the biofilm at different heights (a = 0 µm; b = 1 µm; c = 2 µm; d = 3 µm). (e) Cross section of the biofilm.

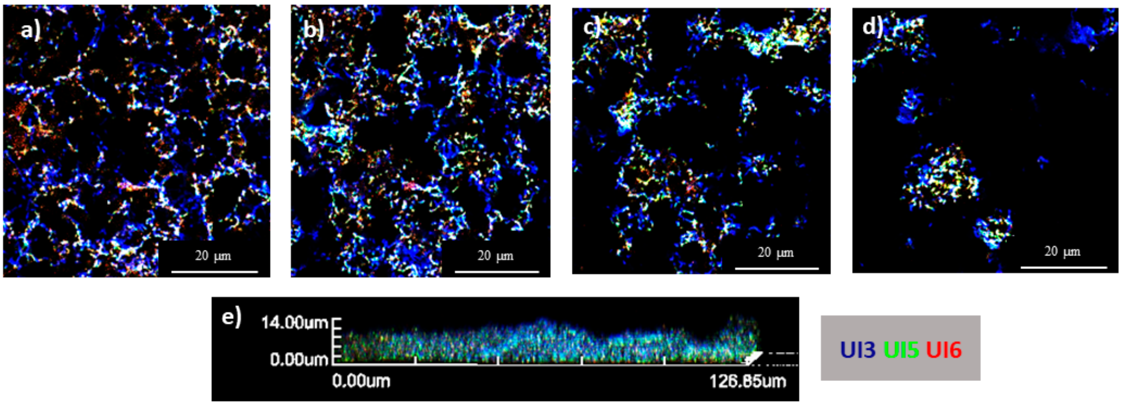

Figure S6. Tri-dimensional organization of 48 h aged biofilm formed in AUM and in polystyrene coupons by a consortium of three *E. coli* strains (UI3 - blue, UI5 - green and UI6 - red). (a) Examples of CLSM images obtained of the layers within the biofilm at different heights (a = 0 µm; b = 2 µm; c = 4 µm; d = 6 µm). (e) Cross section of the biofilm.
